# Supplementary material for: Flexible inference in heterogeneous and attributed multilayer networks
Source: PNAS Nexus. 2025 Jan 9;4(1):pgaf005. doi: 10.1093/pnasnexus/pgaf005 (PMC11756377; doi:10.1093/pnasnexus/pgaf005)
Supplement: pgaf005_Supplementary_Data [file pgaf005_supplementary_data.pdf]

Flexible inference in heterogeneous and attributed multilayer networks

Martina Contisciani, Marius Hobbhahn, Eleanor A. Power, Philipp Hennig and Caterina De Bacco

Corresponding Authors: Martina Contisciani, Caterina De Bacco.
E-mail: martina.contisciani@tuebingen.mpg.de, caterina.debacco@tuebingen.mpg.de

This PDF file includes:

Supporting text
Figs. S1 to S6
Tables S1 to S2
SI References

Supporting Information Text

1. Modelling categorical node metadata

Eq. (5) in the main text describes the general formulation of the expected value of an attribute x for node i. However, when the attribute x is categorical, the expression becomes more intricate because it has to account for the total number of attribute categories Z. In this case, $\boldsymbol{\pi_{ix}}(\boldsymbol{\Theta}) = [\pi_{ixz}(\boldsymbol{\Theta})]_{z\in[1,Z]}$ and $\boldsymbol{H_{kx}} = [H_{kxz}]_{z\in[1,Z]}$ are Z-dimensional vectors. Within this framework, H_{kxz} explains how much information from the z-th category of attribute x is used to create the k-th community, and $\pi_{ixz}(\boldsymbol{\Theta})$ follows the modelling approach outlined in (1):

$$\pi_{ixz}(\boldsymbol{\Theta}) \approx \frac{1}{2}\sum_{k=1}^{K}(U_{ik} + V_{ik})\, H_{kxz}\,. \qquad [1]$$

2. Model settings and hyperparameters choice

We employ PIHAM consistently, maintaining the same configurations and hyperparameters across all experiments. The only variation lies in the choice of the likelihood function, customized to match the data types under examination. Specifically, we adopt Bernoulli distributions for binary information, Poisson distributions for nonnegative discrete data, Gaussian distributions for real values, and Categorical distributions for categorical data.

We set the prior distributions as standard normal distributions $\mathcal{N}(0, 1)$, serving as shrinkage regularization. Indeed, due to the complexity of the data, the objective function may become non-identifiable, thus requiring the enforcement of concavity and differentiability. The selection of these priors accommodates this necessity. On the contrary, for parameter initialization, we opt for wider normal distributions $\mathcal{N}(0, 9)$ to facilitate exploration of various initial points.

PIHAM performs inference using the gradient-based method Automatic Differentiation, and in our implementation we employs the Adam optimizer to iteratively evaluate derivatives of the log-posterior. We set the learning rate of the optimizer equal to 0.5 and run the optimization procedure for 2000 iterations, maintaining a tolerance threshold of 10^{-8}. Furthermore, we execute the algorithm 50 times, each time starting from a different random initialization, and output the parameters corresponding to the realization with the highest objective function.

3. Comparison with existing methods in a homogeneous scenario

Data generation. We construct directed attributed multilayer networks following a procedure similar to that described in (1). Initially, we generate interactions using a multilayer mixed-membership stochastic block model (2). Subsequently, we assign node metadata, ensuring a 50% match with the node communities, while the remaining ones are made randomly. We set a configuration with $N = 500$ nodes, $L = 2$ layers of which one being assortative and the other disassortative, a categorical attribute with $Z = 3$ categories, and $K = 3$ overlapping communities. Networks are generated with increasing average degrees $\langle k\rangle \in \{10, 15, 20, \ldots, 50\}$, producing 20 independent samples for each $\langle k\rangle$ value. To generate the membership matrices $\boldsymbol{U}$ and $\boldsymbol{V}$, we initially assign equal-size unmixed group memberships and then introduce the overlapping for 20% of the nodes. The correlation between $\boldsymbol{U}$ and $\boldsymbol{V}$ is set equal to 0.1, with entries drawn from a Dirichlet distribution with parameter $\alpha = 0.1$. The affinity matrix $\boldsymbol{W^1}$ exhibits an assortative block structure with main probabilities $p_1 = \langle k\rangle K/N$ and secondary probabilities $p_2 = 0.1\, p_1$. Conversely, the affinity matrix $\boldsymbol{W^2}$ is generated using a disassortative block structure with off-diagonal probabilities $p_1 = \langle k\rangle K/N$ and diagonal probabilities $p_2 = 0.1\, p_1$. Self-loops are removed, and sparsity is preserved.

The resulting networks depict a simpler scenario characterized by homogeneous layers with nonnegative discrete weights and a single categorical attribute. Such scenario is crucial for testing our model against existing methods.

Experiment details. For comparison, we use MTCOV (1), a probabilistic model that assumes overlapping communities as the main mechanism governing both interactions and node attributes. This model is specifically tailored to handle categorical attributes and nonnegative discrete weights, and employs an EM algorithm for parameter inference. We run MTCOV 50 times with different random initializations, maintaining the same tolerance as in our implementation. In addition, we set the maximum number of EM steps before termination at 500, and the threshold for declaring convergence based on the consecutive updates respecting the tolerance equal to 15. Lastly, we fix the scaling hyperparameter $\gamma = 0.5$, reflecting the matching constraint imposed in the synthetic data generation.

We assess the performance of PIHAM and MTCOV in both prediction and community detection tasks. Specifically, we evaluate their predictive capabilities using a 5-fold cross-validation routine, in which the dataset is split into five equal-size groups (folds), selected uniformly at random. The models are then trained on four of these folds (training set), covering 80% of the triples (i, j, ℓ) and 80% of the categorical vector entries, to learn their parameters. Next, we evaluate the models' performance on the held-out group (test set). This process is repeated five times by varying the test set, resulting in five trials per iteration. As performance metrics, we use the Area Under the Curve (AUC) for the edge prediction, which represents the probability that a randomly selected edge has a higher expected value than a randomly selected non-existing edge, and accuracy for covariate prediction.

To evaluate the methods' performance in recovering communities, we first need to transform the inferred memberships to match the parameter space of the planted communities. Indeed, the ground truth mixed-memberships are represented as normalized vectors summing to 1, whereas our inferred parameters belong to the real-space. As discussed in the section

"Parameter space and transformations" of the main text, two approaches can be adopted for such alignment: i) applying a softmax transformation to the point estimates $\hat{\boldsymbol{\mu}}^{\boldsymbol{\theta}}$, or ii) employing the LM technique to obtain Dirichlet posterior distributions and utilizing a suitable statistic of these, where the mean serves as a viable option. In this experiment, we use both methods. For assessing performance, we utilize the Cosine Similarity (CS), a metric adept at capturing both hard and mixed-membership communities, ranging from 0 (indicating no similarity) to 1 (denoting perfect recovery). We compute the average cosine similarities of both membership matrices $\boldsymbol{U}$ and $\boldsymbol{V}$, and then average them across the nodes.

Results. The results of both models in prediction and community detection tasks are depicted in Fig. S1. While MTCOV is expected to exhibit better performance due to its close alignment with the generative process underlying the synthetic data, PIHAM demonstrates comparable performance across all tasks despite its broader framework. This similarity is particularly notable in scenarios featuring denser networks. Indeed, our model, by treating all information equally, may face challenges in very sparse networks when relying only on a single covariate. Conversely, this is not an issue for MTCOV as it utilizes a linear combination of node and edge information, leveraging node covariates to address network sparsity.

An additional consideration is the choice of transformation used to compare the inferred communities with the planted ones. Both options yield similar results, as shown in Fig. S1C. The slight discrepancy between the two arises from the Dirichlet mean providing slightly more mixed-memberships.

Overall, these findings collectively suggest that PIHAM remains a valid approach even in less heterogeneous scenarios, demonstrating its ability to effectively compete with ad-hoc existing methods.

4. Validation on heterogeneous data

Data generation. We construct directed, heterogeneous, and attributed multilayer networks using the framework of PIHAM. Initially, we draw the latent variables $\boldsymbol{\Theta} = (\boldsymbol{U}, \boldsymbol{V}, \boldsymbol{W}, \boldsymbol{H})$ from Gaussian distributions with specified hyperparameters, and then generate $\boldsymbol{A}$ and $\boldsymbol{X}$ according to the data types, following Eqs. (2)-(7) in the main text. We configure the networks with $L = 3$ heterogeneous layers: one with binary interactions, the second with nonnegative discrete weights, and the third with real values. Additionally, each node is associated with three covariates: one categorical with $Z = 4$ categories, one with nonnegative discrete values, and the last with real values. Networks are generated with increasing number of nodes $N \in \{100, 200, \ldots, 1000\}$, and varying number of overlapping communities $K \in \{3, 4, 5\}$. For each combination (N, K), we generate 20 different samples. To generate the membership matrices $\boldsymbol{U}$ and $\boldsymbol{V}$, we assign equal-size group memberships and draw the entries of the matrices from distributions with different means, according to the group the nodes belong to. Specifically, $u_{ik} \sim \mathcal{N}(2, 0.04)$ if i is associated with group k, otherwise $u_{ik} \sim \mathcal{N}(-1, 0.04)$. Similarly, $v_{ik} \sim \mathcal{N}(2, 0.09)$ if i is associated with group k, otherwise $v_{ik} \sim \mathcal{N}(-1, 0.09)$. The affinity tensor $\boldsymbol{W}$ exhibits an assortative block structure in each layer, with diagonal entries following normal distributions with zero mean and $\sigma = 0.45$, and off-diagonal entries drawn from normal distributions with $\mu = -4$ and $\sigma = 0.45$. The community-covariate matrix $\boldsymbol{H}$ is set to maintain coherence between node interactions and covariates, avoiding additional noise in the data. For the categorical variable, $H_{kxz} \sim \mathcal{N}(0.5 + k, 0.04)$ if $k = z$, otherwise $H_{kxz} \sim \mathcal{N}(0, 0.04)$. When $K = 3$ or $K = 5$, we set $H_{kx4} \sim \mathcal{N}(0.2, 0.04)$ or $H_{5xz} \sim \mathcal{N}(0.2, 0.04)$, respectively. The nonnegative discrete attribute is generated according to $H_{kx} \sim \mathcal{N}(1.5 \times \frac{k+2}{3}, 0.01)$, while the covariate with real values is constructed with $H_{kx} \sim \mathcal{N}(4 + (1 - k) \times 3, 0.04)$.

The resulting networks represent a general scenario featuring heterogeneous interactions and node covariates, which is essential to validate our approach and demonstrate its flexibility.

Experiment details. We assess the performance of PIHAM by evaluating its predictive capabilities through a 5-fold cross-validation routine. In this approach, the dataset is randomly divided into five equal-sized groups (folds), and the model is trained on four of them (the training set), which include 80% of the triples (i, j, ℓ) and 80% of the entries of each attribute vector, to learn its parameters. The performance of the model is then evaluated on the remaining fold (the test set). This process is repeated five times, each time with a different fold as the test set, resulting in five trials per iteration. For performance metrics, we use different measures depending on the type of information being evaluated. For binary interactions, we use AUC, which ranges from 0 to 1, with 0.5 representing the random baseline. For nonnegative discrete data, we use the Maximum Absolute Error (MAE), and for real values, we use the Root Mean Squared Error (RMSE). In both cases, lower values indicate better performance. Additionally, for categorical attribute predictions, we use accuracy, which ranges from 0 to 1, with 1 indicating perfect recovery.

5. Interpretation of posterior estimates

Experiment details. Interpreting posterior distributions can be challenging, especially with large datasets. To address this, we propose two different approaches to summarize the inferred results.

First, we employ a metric to quantify the area of overlap between distributions. We use the method proposed in (3), which defines the integrated absolute error (IAE) between two distributions as:

$$\mathrm{IAE} = \int_{-\infty}^{\infty} \left| \, p_1(x) - p_2(x) \, \right| dx \,, \qquad [2]$$

with IAE $\in [0, 2]$. When p_1 and p_2 completely overlap, the difference between them is a line in zero, and the IAE is 0. Conversely, if there is no overlap, the IAE is 2, which is the sum of the integrals of the two distributions. To normalize this metric to the range $[0, 1]$, we define:

$$\text{Overlap} = 1 - \frac{1}{2}\,\text{IAE}\,. \qquad [3]$$

In this case, an Overlap of 0 indicates no overlap between the distributions, while an Overlap of 1 represents perfect matching. We compute this measure between every pair of distributions for each node and then calculate the average.

Computing the Overlap for many communities can be computationally expensive due to the need to evaluate all pairwise combinations. As an alternative, we use the L_2-barycenter distribution, which represents a weighted average of the node-community distributions (4, 5). This approach simplifies the problem by focusing on a single distribution per node instead of K different ones. We calculate this distribution using the `POT Python package` (6), and we quantify it by computing its variance (σ^2) using the trapezoidal rule to approximate the integral. Higher values indicate nodes with harder memberships, as the barycenter is more spread due to the individual distributions being more distant from each other. Conversely, lower variance suggests more overlap among the distributions, indicating a more mixed-membership scenario.

6. Analysis of a social support network of a rural Indian village

Data pre-processing. We analyze a real-world dataset describing a social support network within a village in Tamil Nadu, India, referred to as "Aḻakāpuram" (7, 8). The data were collected in 2013 through surveys, in which adult residents were asked to nominate individuals who provided various types of support. In our analysis, we consider six different binary support questions, each forming a layer in the network, and we exclude individuals without any of these interactions. Details on these layers, including the number of edges and the average degree, are provided in Table S1, while a visual representation can be found in Fig. S2. Additionally, we construct a seventh layer that incorporates the geographical distance between individuals' households. Specifically, we define the entries of this layer as $A^7_{ij} = \frac{1}{\sqrt{1+d_{ij}}}$, where d_{ij} is the distance between the households of individuals i and j, and we set $A^7_{ii} = 0$. Note that higher values indicate closer proximity, while lower values represent greater distances. The resulting adjacency tensor is then represented as $\boldsymbol{A} = \{\boldsymbol{A}^{\boldsymbol{\ell}} \in \{0,1\}^{N\times N}\ \forall \ell \in [1,6], \boldsymbol{A}^{\boldsymbol{7}} \in \mathbb{R}_+{}^{N\times N}\}$.

In addition, several attributes were collected, including information like gender, age, and caste, among others. For our analysis, we focus on caste, religion, and years of education, as ethnographic work and previous analyses (8, 9) suggest these attributes significantly influence how villagers relate to one another. Specifically, caste is a categorical attribute with $Z_{caste} = 14$ categories, religion has $Z_{religion} = 3$ categories, and years of education are represented as nonnegative discrete values $\boldsymbol{X}_{\boldsymbol{\cdot 3}} \in \mathbb{N}_0^N$.

The resulting heterogeneous attributed multilayer network comprises $N = 419$ nodes, $L = 7$ layers, and $P = 3$ node attributes.

Results. To determine the number of communities K, we employ a 5-fold cross-validation procedure for $K \in [1, 10]$ and select the value that exhibits the optimal performance. Similar to the synthetic experiments, for a given K, we train the model on four folds (training set), which include 80% of the triples (i, j, ℓ) and 80% of the entries of each attribute vector, to learn its parameters. The model's performance is then evaluated on the remaining fold (the test set). This process is repeated five times, each with a different fold as the test set, resulting in five trials per iteration. We evaluate prediction performance in the first six binary layers using the AUC, ranging from 0 to 1, with 0.5 indicating the random baseline. For the seventh layer containing real values, we use the RMSE, where lower values indicate better performance. Additionally, we assess prediction performance for the attributes using accuracy for caste and religion, which ranges from 0 to 1 (with 1 indicating perfect recovery), and the MAE for years of education, with lower values indicate better performance. The results are displayed in Table S2. In our experiments, we set $K = 6$ as it achieves the best performance across most prediction metrics. Note that, summarizing and evaluating results in a heterogeneous setting using a single metric is challenging, as discussed in the section "Validation on heterogeneous data" of the main text. The results in Table S2 also demonstrate that PIHAM achieves robust outcomes with the chosen fixed value of K.

We further assess whether the model is a good fit to the data by conducting a posterior–predictive test (10, 11), where we compare the input data $(\boldsymbol{A}, \boldsymbol{X})$ to synthetic data $(\tilde{\boldsymbol{A}}, \tilde{\boldsymbol{X}})$ generated by the fitted model. A well-fitted model should produce synthetic data that closely match the original input. The posterior-predictive distribution is defined as

$$P(\tilde{\boldsymbol{A}}, \tilde{\boldsymbol{X}} \mid \boldsymbol{A}, \boldsymbol{X}) = \int_{\boldsymbol{\Theta}} P(\tilde{\boldsymbol{A}}, \tilde{\boldsymbol{X}} \mid \boldsymbol{\Theta})\, P(\boldsymbol{\Theta} \mid \boldsymbol{A}, \boldsymbol{X})\, d\boldsymbol{\Theta}\,, \qquad [4]$$

and samples from this distribution are obtained by first drawing $\boldsymbol{\Theta}$ from the posterior distributions $\mathcal{N}(\boldsymbol{\theta}; \hat{\boldsymbol{\mu}}^{\boldsymbol{\theta}}, \hat{\boldsymbol{\Sigma}}^{\boldsymbol{\theta}})$, and then using these parameters to create new synthetic data. We apply the likelihood described in the previous subsection, fixing the standard deviation to 0.1 for the Gaussian layer, and generate 500 independent synthetic datasets. To evaluate performance, we test whether two samples from the posterior-predictive distribution are generally more, equally, or less distant from each other than a sample from the posterior-predictive distribution compared to the input data (10). We measure the distance using different metrics depending on the data type: log-loss for the six binary layers; RMSE for the seventh layer with real values, (1 − accuracy) for categorical attributes, and MAE for the attribute representing years of education. The results in Fig. S3 show that the discrepancies between synthetic data samples consistently exceed those between the observed data and synthetic samples, indicating that PIHAM provides a good fit for the data.

To provide a qualitatively interpretation of the inferred results, Fig. 4 in the main text shows the inferred out-going communities $\hat{\boldsymbol{U}}$. Here, we present additional visualizations for other model parameters. In particular, Fig. S4 displays the inferred $K \times Z_{caste}$-dimensional matrix $\hat{\boldsymbol{H}}_{\boldsymbol{\cdot 1}}$, which explains the contributions of each caste category to the formation of the k-th community. Panel B presents the inferred posterior distributions $\hat{H}_{k1z} \sim \mathcal{N}(\hat{H}_{k1z}; \hat{\mu}^{H}_{k1z}, (\hat{\sigma}^{H}_{k1z})^2)$, with different colors representing distinct caste categories. Panel A, instead, shows the softmax transformation of the MAP estimates $\hat{\boldsymbol{\mu}}^{\boldsymbol{H}}_{\boldsymbol{k1}}$ for easier interpretation. Note that, the matrix is transposed in the plot, so that each column sums to 1, and the y-axis lists the caste categories. From this figure, we observe that the first and second communities predominantly consist of nodes from to the Yātavar and Paṟaiyar castes, respectively. Similarly, K_3 comprises nodes from the Kulālar and Maṟavar castes, while communities K_4, K_5, and K_6 are predominantly composed by nodes from the Paḷḷar caste, which is also the most represented caste in the dataset. Furthermore, Fig. S5 shows the inferred posterior distributions of the community-covariate vector related to years of education, $\hat{H}_{k3} \sim \mathcal{N}(\hat{H}_{k3}; \hat{\mu}^{H}_{k3}, (\hat{\sigma}^{H}_{k3})^2)$. From this figure, it is evident that this attribute plays a more significant role in determining K_6, as its distribution has a notably higher mean compared to the distributions of the other communities. Lastly, Fig. S6 displays the affinity tensor $\hat{\boldsymbol{W}}$, which explains the edge density between different community pairs in the various layers. To improve visualization clarity, we apply a logistic transformation to the MAP estimates $[\hat{\mu}^{\ell}_{kq}]^{W}$. This figure suggests that the different layers predominantly exhibit an assortative structure, where nodes tend to interact more with individuals within the same community than with those from different communities. However, we notice some variations for certain layers. For instance, L_2 (help finding a job) has few non-zero diagonal values, suggesting that this type of support sometimes requires seeking out individuals in different communities. Moreover, L_7, corresponding to the geographical distance between nodes, has several off-diagonal entries, particularly for communities K_4, K_5, and K_6, suggesting a weakened effect for physical proximity for those communities. Taken together, these findings suggest that PIHAM utilizes all the input information to infer partitions that effectively integrate all of them in a meaningful manner. In addition, the inferred affinity matrices illustrate how different layers can exhibit different community structures, a diversity that can be captured by our model.

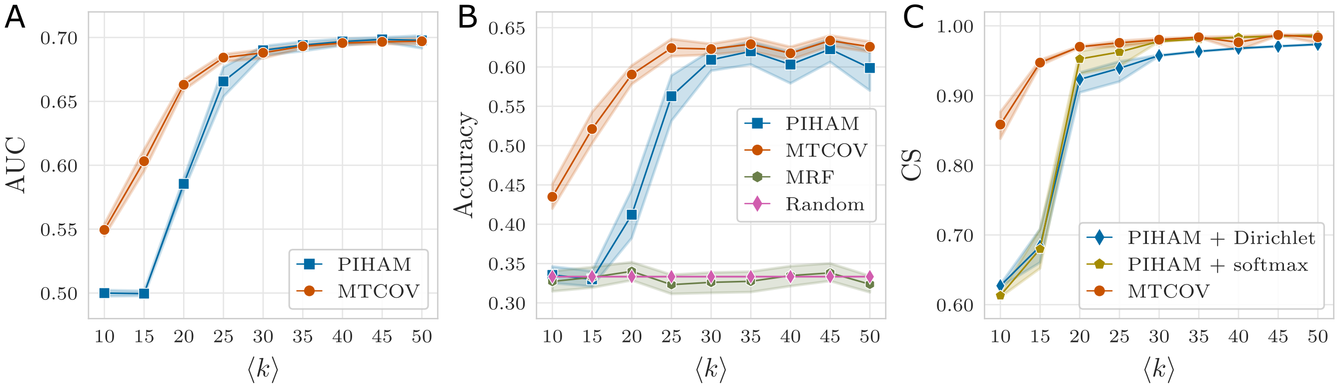

Fig. S1. Prediction and community detection performance on synthetic data. We analyze synthetic attributed multilayer networks with $N = 500$ nodes, $L = 2$ layers (one being assortative and the other disassortative), a categorical attribute with $Z = 3$ categories, $K = 3$ overlapping communities, and increasing average degrees $\langle k \rangle \in \{10, 15, 20, \dots, 50\}$. The results represent averages and confidence intervals over 20 independent samples. For prediction tasks, we employ a 5-fold cross-validation procedure. The evaluation metrics include (A) the AUC for edge prediction, with a baseline of 0.5 corresponding to random choice, and (B) accuracy for covariate prediction. Here, MRF represents a baseline given by the predictions obtained from the maximum frequency in the training set, while Random denotes the uniform random probability over Z. (C) Community detection performance is assessed using Cosine Similarity (CS). As inferred point estimates, we consider both the mean of transformed Dirichlet posterior distributions and the $\mathrm{softmax}$ transformation of $\hat{\boldsymbol{\mu}}^{\boldsymbol{\theta}}$. Overall, PIHAM exhibits comparable performance to MTCOV across all tasks despite its broader framework, especially in scenarios involving denser networks.

Table S1. Summary statistics for the first six binary layers of the "Al̠akāpuram" social support network. E denotes the number of edges, while $\langle k \rangle$ represents the average degree. A visual representation of these layers can be found in Fig. S2.

Layer	Description	E	$\langle k \rangle$
L_1	Talk about important matters	880	4.2
L_2	Help finding a job	437	2.1
L_3	Help with physical tasks	758	3.6
L_4	Borrow household items from	876	4.2
L_5	Ask for money	386	1.8
L_6	Talk to for pleasure	824	3.9

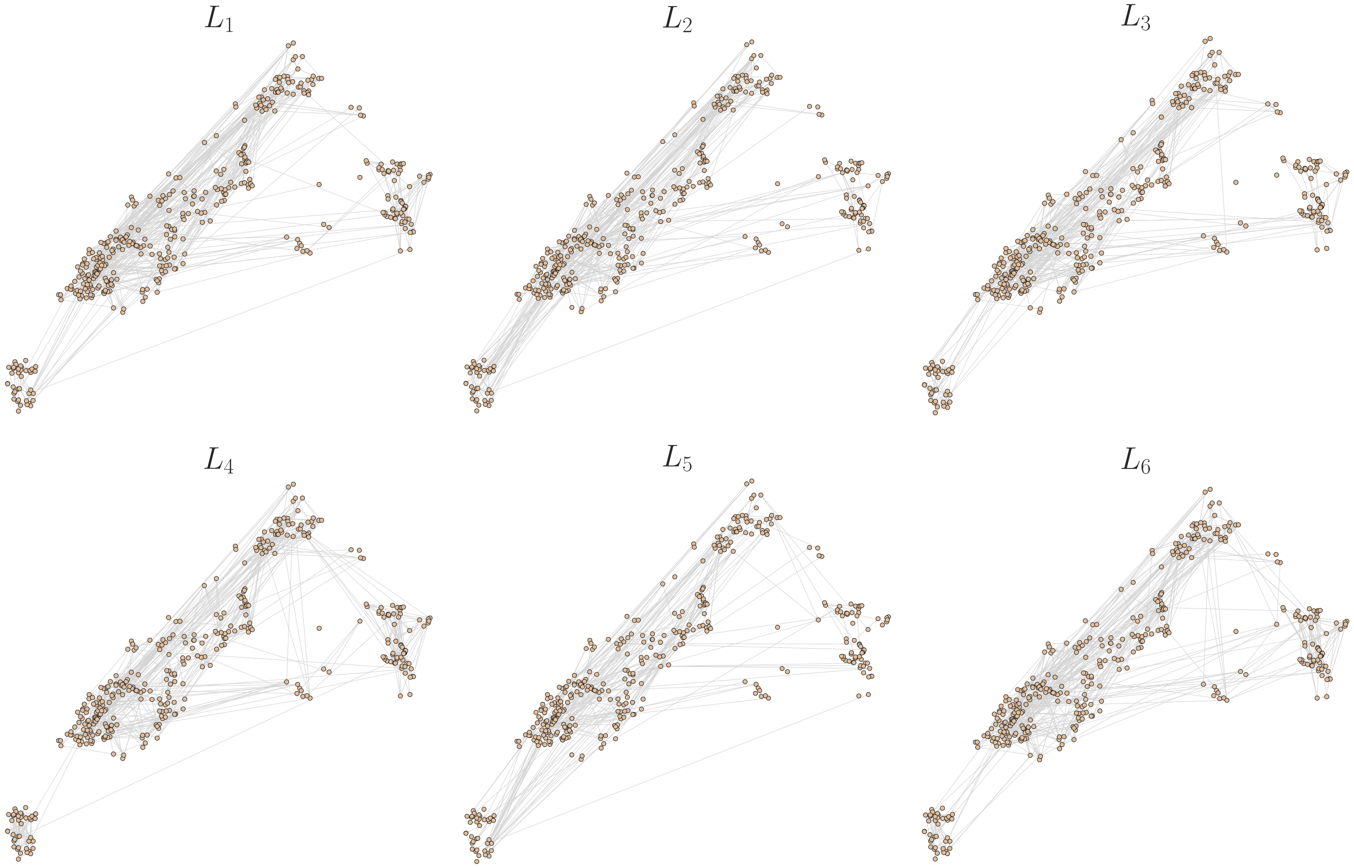

Fig. S2. Visual representation of the first six binary layers of the "Aḻakāpuram" social support network. Details on these layers, including the information encoded, the number of edges, and the average degree, are provided in Table S1. The position of the nodes reflects the geographical distance between individuals' households.

Table S2. Prediction performance on the "Aḻakāpuram" social support network. For a given number of communities K, we employ a 5-fold cross-validation procedure and report averages and standard deviations over the five trials, each using a different fold as the test set. We evaluate prediction performance in the first six binary layers using the Area Under the Curve (AUC) and for the seventh layer containing real values using the Root Mean Squared Error (RMSE). Additionally, we assess prediction performance for the attributes using accuracy for caste ($X_{\cdot 1}$) and religion ($X_{\cdot 2}$), and the Maximum Absolute Error (MAE) for years of education ($X_{\cdot 3}$), represented as nonnegative discrete values. The baselines are omitted for brevity. In our experiments, we set $K = 6$ as it achieves the best performance across most prediction metrics, and overall, PIHAM demonstrates robust outcomes with the chosen fixed value of K.

K	AUC ($[\boldsymbol{A}^{\ell}]_{\ell \in [1,6]}$)	RMSE ($\boldsymbol{A}^{7}$)	Accuracy ($\boldsymbol{X}_{\cdot 1}$)	Accuracy ($\boldsymbol{X}_{\cdot 2}$)	MAE ($\boldsymbol{X}_{\cdot 3}$)
1	0.575 ± 0.007	0.096 ± 0.003	0.55 ± 0.05	0.84 ± 0.04	4.3 ± 0.3
2	0.717 ± 0.009	0.096 ± 0.003	0.55 ± 0.04	0.84 ± 0.04	4.3 ± 0.2
3	0.73 ± 0.01	0.096 ± 0.003	0.55 ± 0.05	0.84 ± 0.04	$\mathbf{4.3 \pm 0.1}$
4	$\mathbf{0.77 \pm 0.01}$	0.093 ± 0.003	0.58 ± 0.05	0.84 ± 0.03	4.4 ± 0.1
5	0.77 ± 0.02	0.088 ± 0.005	0.63 ± 0.06	0.87 ± 0.06	4.4 ± 0.1
6	0.76 ± 0.01	$\mathbf{0.086 \pm 0.003}$	$\mathbf{0.70 \pm 0.07}$	$\mathbf{0.87 \pm 0.02}$	4.4 ± 0.1
7	0.74 ± 0.01	0.089 ± 0.003	0.63 ± 0.04	0.85 ± 0.04	4.4 ± 0.1
8	0.73 ± 0.01	0.095 ± 0.003	0.27 ± 0.06	0.83 ± 0.04	4.4 ± 0.1
9	0.72 ± 0.01	0.095 ± 0.003	0.20 ± 0.04	0.84 ± 0.04	4.4 ± 0.1
10	0.72 ± 0.01	0.095 ± 0.003	0.2 ± 0.2	0.84 ± 0.04	4.4 ± 0.2

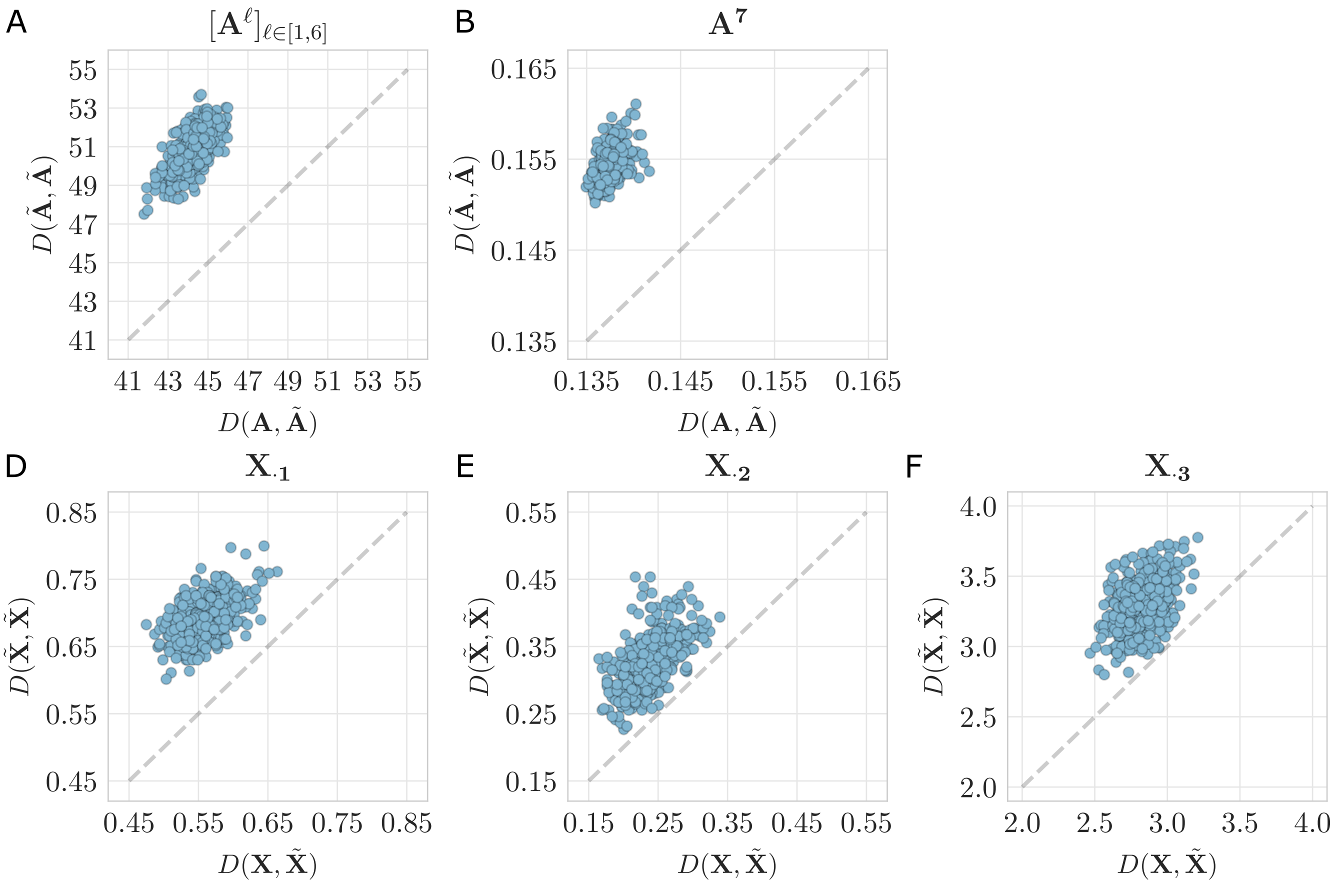

Fig. S3. Goodness of fit test for the "Aḻakāpuram" social support network. We generate 500 independent synthetic datasets using the fitted model and compare them to both the input data and other synthetic samples. Each dot represents one posterior-predictive sample, showing the distance between this sample and the input data on the horizontal axis, and a randomly selected synthetic sample on the vertical axis. Performance is evaluated using different metrics D depending on the data type: log-loss for binary interactions (A), RMSE for real values (B), $(1 - \text{accuracy})$ for categorical attributes (D, E), and MAE for nonnegative discrete values (F). The differences between synthetic data samples consistently exceed those between the observed data and synthetic samples, as all points lie above the diagonal, indicating that PIHAM provides a good fit for the data.

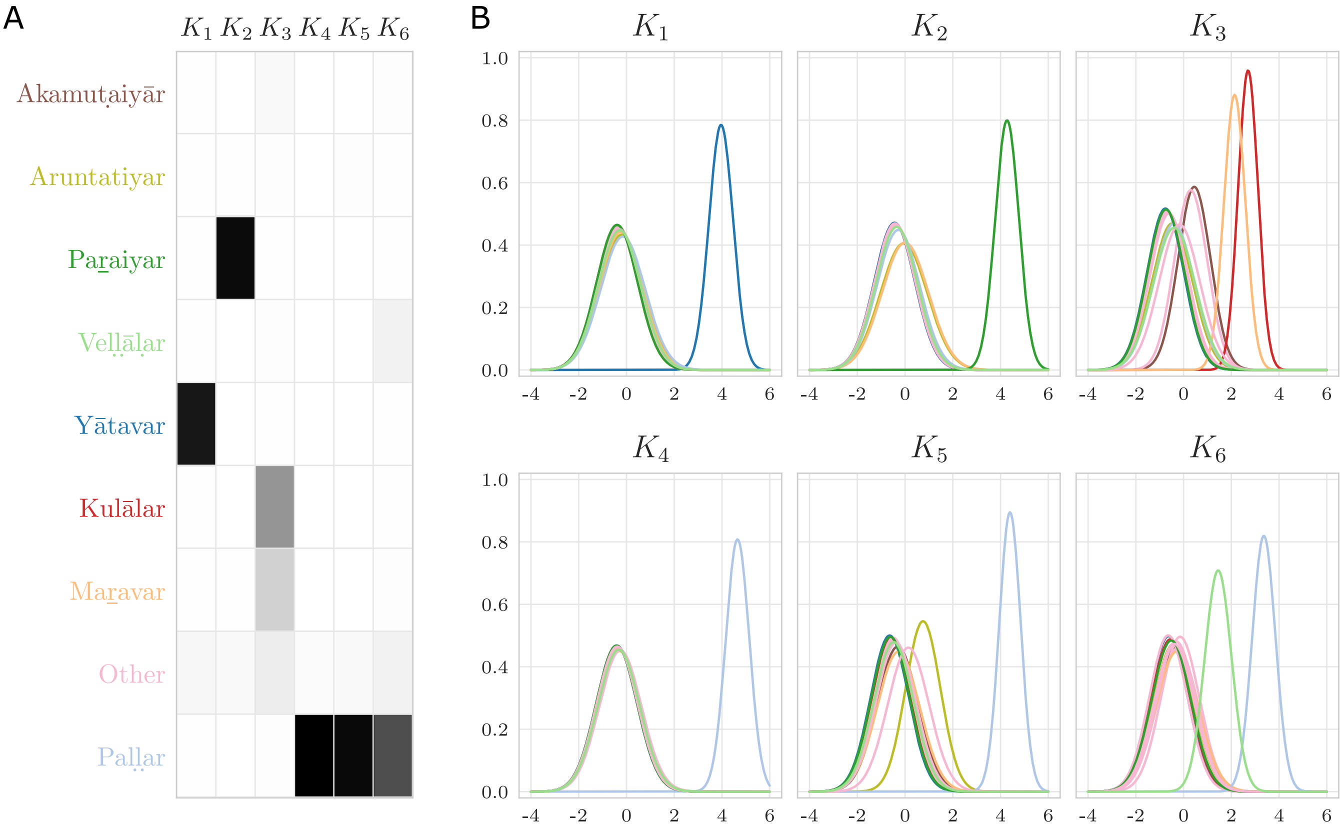

Fig. S4. Inference of the community-caste parameter in the "Aḻakāpuram" social support network. We display the inferred $K \times Z_{caste}$-dimensional matrix $\hat{\boldsymbol{H}}_{\cdot \boldsymbol{1}}$, which explains the contributions of each caste category to the formation of the k-th community. For privacy reasons, nodes belonging to castes with fewer than five individuals are aggregated into an "Other" category. (A) Transformation of the MAP estimates $\hat{\boldsymbol{\mu}}^{\boldsymbol{H}}_{\boldsymbol{k1}}$ inferred by PIHAM using the $\mathrm{softmax}$ function. In this plot, the matrix is transposed, so that each column sums to 1, and the y-axis lists the caste categories. (B) Inferred posterior distributions $\hat{H}_{k1z} \sim \mathcal{N}(\hat{H}_{k1z}; \hat{\mu}^{H}_{k1z}, (\hat{\sigma}^{H}_{k1z})^2)$, with different colors representing distinct caste categories. The first and second communities predominantly consist of nodes from to the Yātavar and Paṟaiyar castes, respectively. K_3 comprises nodes from the Kulālar and Maṟavar castes, while communities K_4, K_5, and K_6 are predominantly composed by nodes from the Paḷḷar caste.

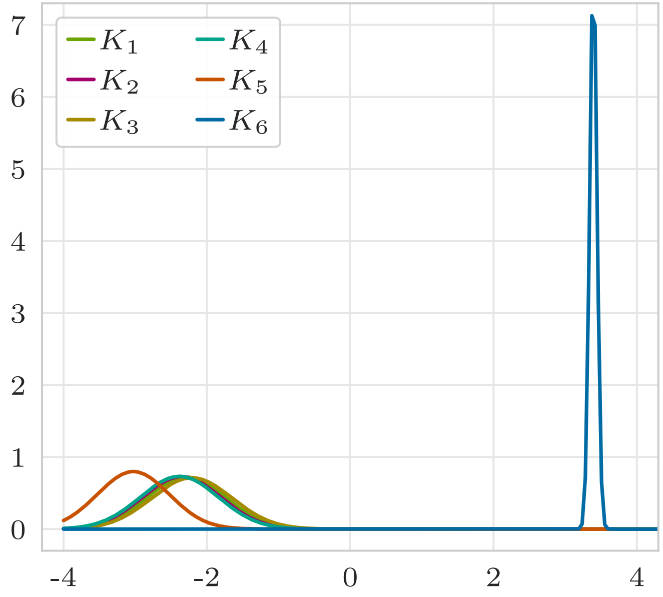

Fig. S5. Inference of the community-education parameter in the "Aḻakāpuram" social support network. We display the inferred posterior distributions $\hat{H}_{k3} \sim \mathcal{N}(\hat{H}_{k3}; \hat{\mu}^H_{k3}, (\hat{\sigma}^H_{k3})^2)$, which explain how the attribute related to years of education is distributed among the K communities. The distribution of the attribute in K_6 has a notably higher mean compared to the distributions of the other communities, suggesting that $\boldsymbol{X}_{\cdot 3}$ plays a significant role in determining this community.

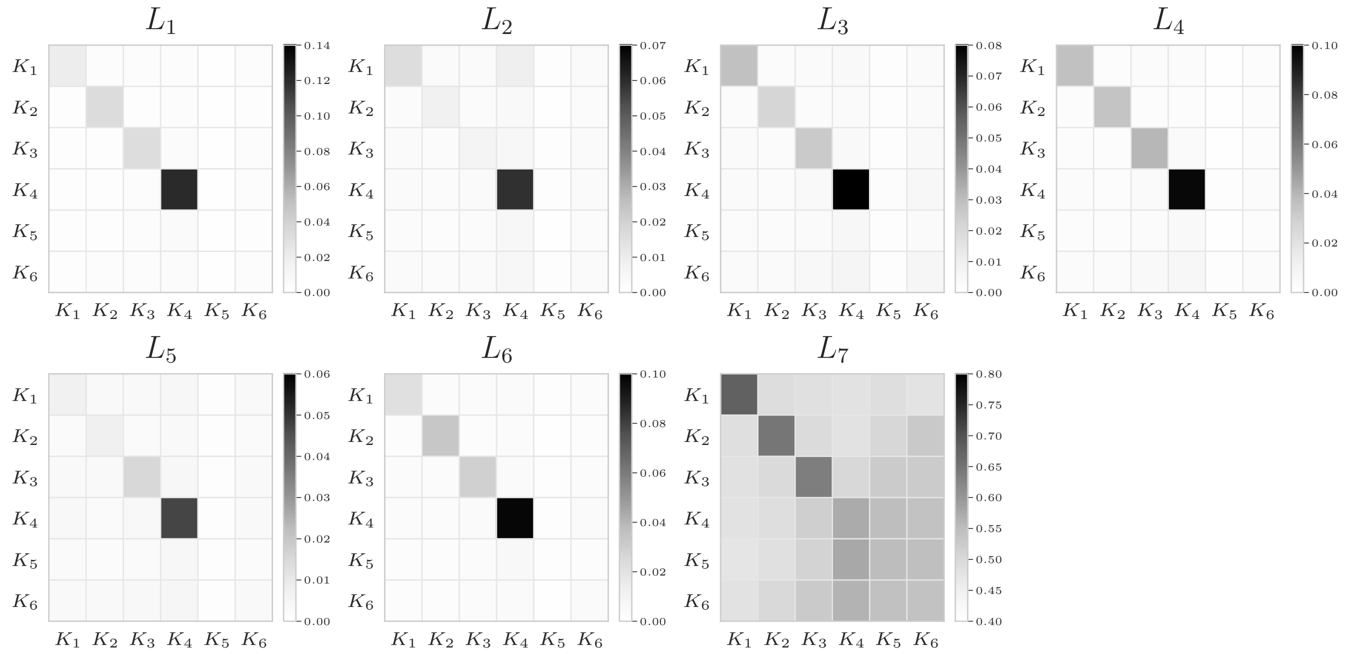

Fig. S6. Inference of the affinity tensor in the "Al̲akāpuram" social support network. We display the MAP estimates of the inferred $\hat{\boldsymbol{W}}$, which explain the edge density between different community pairs in the various layers. To improve visualization clarity, we apply a $\mathrm{logistic}$ transformation to the MAP estimates $[\hat{\mu}^{\ell}_{kq}]^{W}$. The different layers predominantly exhibit an assortative structure, with some variations for certain layers. For instance, L_2 (help finding a job) has few non-zero diagonal values, suggesting that this type of support sometimes requires seeking out individuals in different communities. Moreover, L_7, corresponding to the geographical distance between nodes, has several off-diagonal entries, particularly for communities K_4, K_5, and K_6, suggesting a weakened effect for physical proximity for those communities.

References

1. M Contisciani, EA Power, C De Bacco, Community detection with node attributes in multilayer networks. *Sci. Reports* **10**, 15736 (2020).
2. C De Bacco, EA Power, DB Larremore, C Moore, Community detection, link prediction, and layer interdependence in multilayer networks. *Phys. Rev. E* **95**, 042317 (2017).
3. MP Wand, JT Ormerod, SA Padoan, R Frühwirth, Mean Field Variational Bayes for Elaborate Distributions. *Bayesian Analysis* **6**, 847 – 900 (2011).
4. JD Benamou, G Carlier, M Cuturi, L Nenna, G Peyré, Iterative bregman projections for regularized transportation problems. *SIAM J. on Sci. Comput.* **37**, A1111–A1138 (2015).
5. CL Coz, A Tantet, R Flamary, R Plougonven, A barycenter-based approach for the multi-model ensembling of subseasonal forecasts. *arXiv preprint arXiv:2310.17933* (2023).
6. R Flamary, et al., Pot: Python optimal transport. *J. Mach. Learn. Res.* **22**, 1–8 (2021).
7. EA Power, *Building bigness: Religious practice and social support in rural South India.* (Stanford University), (2015).
8. EA Power, Social support networks and religiosity in rural south india. *Nat. Hum. Behav.* **1**, 0057 (2017).
9. EA Power, E Ready, Building bigness: Reputation, prominence, and social capital in rural south india. *Am. Anthropol.* **120**, 444–459 (2018).
10. A Gelman, XL Meng, H Stern, Posterior predictive assessment of model fitness via realized discrepancies. *Stat. sinica* pp. 733–760 (1996).
11. A Gelman, et al., *Bayesian Data Analysis.* (CreateSpace, United States), 3rd ed edition, (2013).
